# Glutamate, Glutamine and GABA Levels in Rat Brain Measured Using MRS, HPLC and NMR Methods in Study of Two Models of Autism

**DOI:** 10.3389/fnmol.2018.00418

**Published:** 2018-11-16

**Authors:** Elzbieta Zieminska, Beata Toczylowska, Dominik Diamandakis, Wojciech Hilgier, Robert Kuba Filipkowski, Rafal Polowy, Jaroslaw Orzel, Michal Gorka, Jerzy Wieslaw Lazarewicz

**Affiliations:** ^1^Mossakowski Medical Research Centre, Polish Academy of Sciences, Warsaw, Poland; ^2^Nalecz Institute of Biocybernetics and Biomedical Engineering, Polish Academy of Sciences, Warsaw, Poland; ^3^Faculty of Electronics and Information Technology, Warsaw University of Technology, Warsaw, Poland; ^4^Faculty of Physics, University of Warsaw, Warsaw, Poland

**Keywords:** ASD animal models, neurotransmitters, MRS, NMR, HPLC, USV

## Abstract

The disorders of the glutamatergic neurotransmission have been associated with pathogenesis of autism. In this study we evaluated the impact of the *in vivo* and *ex vivo* test methodology on measurements of levels of neurotransmitter amino acids in hippocampus of rats for valproic acid- (VPA) and thalidomide- (THAL) induced models of autism. The main goal was to compare the changes in concentrations of glutamate (Glu), glutamine (Gln) and GABA between both autistic groups and the control, measured *in vivo* and *ex vivo* in homogenates. The rat pups underwent three *in vivo* tests: ultrasonic vocalization (USV), magnetic resonance spectroscopy (MRS) and unilateral microdialysis of the hippocampus. Analyses of homogenates of rat hippocampus were performed using high-performance liquid chromatography (HPLC) and nuclear magnetic resonance (NMR) spectroscopy. For the statistical analysis, we performed univariate and multivariate tests. USV test, which is considered in rodents as an indicator of pathology similar to autism, showed decreased USV in VPA and THAL groups. *In vivo* MRS studies demonstrated increases of Glu content in male rat’s hippocampus in VPA and THAL groups, while the microdialysis, which allows examination of the contents in the extracellular space, detected decreases in the basal level of Gln concentrations in VPA and THAL groups. *Ex vivo* HPLC studies showed that levels of Glu, Gln and GABA significantly increased in male rat’s hippocampus in the VPA and THAL groups, while NMR studies showed increased levels of Gln and GABA in the VPA group. Collectively, these results are consistent with the hypothesis suggesting the role of the glutamatergic disturbances on the pathogenesis of autism. For all methods used, the values of measured changes were in the same direction. The orthogonal partial least square discriminant analysis confirmed that both animal models of autism tested here can be used to trace neurochemical changes in the brain.

## Introduction

Autism spectrum disorders (ASD) including autism itself, comprise a group of severe neurodevelopmental disabilities that is revealed during the first 3 years of life. The disorders are characterized by impairments in social interactions and communication as well as restricted interests and repetitive behaviors, sometimes accompanied by self-destructive behaviors (Masi et al., 2017). Occurrence of autism as a disease has been increasing over time, varying depending on country from 2/10000 to 30/10000 (Sadamatsu et al., 2006). Although approximately 10% of autism cases can be attributed to genetic causes, such as fragile X syndrome, remaining cases are idiopathic, lacking established etiology. Most research groups believe that genetic components combined with environmental factors play a significant role in the pathophysiology of idiopathic ASD (Ansel et al., 2016; Ng et al., 2017).

Similar to other diseases of the central nervous system, the proposed pathogenesis of autism seems to have aspects of glutamatergic dysfunction (Rojas, 2014), although this is still not fully confirmed. There are conflicting views whether excessive inhibition or hyperactivity of the glutamatergic system occurs in autism (Carlsson, 1998; Fatemi, 2008). However, it now seems that imbalances between excitation and inhibition contribute to the development and maintenance of ASD (Lee et al., 2017). This assertion is supported by the results of the receptor protein expression studies. There is existing data indicating that in brains of autistic patients, in addition to up-regulation of mGluR5, *N*-methyl-D-aspartate receptors (NMDARs) and glutamate transporters (Rinaldi et al., 2007; Fatemi et al., 2011; Lohith et al., 2013) inhibition of the GABA-ergic neurotransmission (Gogolla et al., 2009; Gaetz et al., 2014) has been observed. Hyperactivity of NMDARs that has been demonstrated in animal models of autism seems to be mediated by mGluR5, probably because of partial reduction caused by mGluR5 antagonists (Carlson, 2012). This provided the basis for the use of NMDAR and mGluR5 antagonists in the experimental treatment of behavioral disorders in ASD patients (Chez et al., 2007; Jacquemont et al., 2011). Important information supporting the glutamate hypothesis of autism also come from genetic research, which showed association between autism and genes for kainate and AMPA receptors, as well as for NMDARs, mGluRs or glutamate transporters (Jacob et al., 2011; Carlson, 2012; Yoo et al., 2012).

The issue of concentrations of the excitatory and inhibitory amino acid neurotransmitters glutamate and GABA, respectively, in the brain of autistic patients and in animal models of autism seems more unclear. Although most studies consistently showed an increase of blood glutamate concentration, studies using proton magnetic resonance spectroscopy (MRS) gave inconsistent information on the concentrations of glutamate (Glu), glutamine (Gln) and GABA, in different regions of the brain of autistic patients (Rojas, 2014; Dickinson et al., 2016). These results come from various laboratories with MRS devices that differ in terms of magnetic field strength (1.5T to 7T), for review see (Ford and Crewther, 2016). Therefore, the aforementioned differences in results of studies may be caused not only by the known variability of the autistic-like phenotypes and the variety of clinical symptoms (Moreno-De-Luca et al., 2013), but also by the methodological diversity.

The goal of our study was to evaluate the impact of *in vivo* and *ex vivo* methodology on measurements of neurotransmitter amino acids in rat hippocampus as applied in studies of chemical models of autism. We also attempted to assess the compatibility of amino acid concentrations changes observed by us with previously published results observed in children with autism using the MRS method. We expect that obtaining consistent results would promote wider use of chemical rat models of autism in studies of the role of glutamatergic disturbances in the pathogenesis of the disease and in testing therapeutic strategies. For each animal, three different analytical methods were applied to measure and compare the changes in concentrations of Glu, Gln, and GABA in rat hippocampus. Two animal models of autism were studied; both were induced by applying teratogenic drugs during the rat fetal period: valproic acid (VPA) or thalidomide (THAL), respectively. Results from the *in vivo* MRS analysis were presented together with the results of HPLC measurements of the amino acid concentration in microdialysates of the hippocampus collected *in vivo*. Total levels of Glu, Gln and GABA in the homogenates of the hippocampi were measured *ex vivo* using HPLC and nuclear magnetic resonance (NMR) spectroscopy. We used ultrasonic vocalization (USV) test to ensure that animals belonging to the experimental groups exhibit autistic behavior.

## Materials and Methods

### Animals

Experiments were performed using Wistar rats (Cmd: (WI)WU) of both sexes. Rats were bred in the Animal Colony of the Mossakowski Medical Research Centre, Polish Academy of Sciences in Warsaw. The animals were given water and were fed *ad libitum* and were kept on a 12-h dark-light cycle at room temperature with constant humidity of approximately 60%. All of the procedures in animal experiments were approved by the Forth Local Ethical Committee in Warsaw (resolution no. 43/2015 of May 22, 2015) and were performed in accordance with the European Community Council Directive of 24 November 1986 (86/609/EEC) and corresponding Polish government regulations concerning animal experimentation. All efforts were made to minimize animal suffering and the number of animals used in testing.

### Animal Models of Autism

**FIGURE 1:**
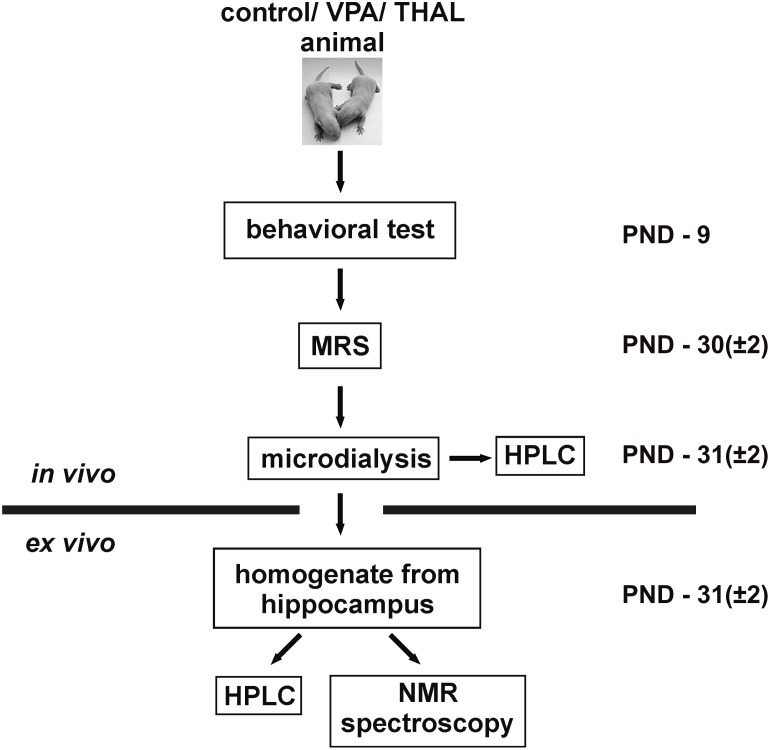
The scheme of the experimental procedure that all animals have undergone.

In our project we used two chemical teratogenic models of autism. Female rats on the 11th day of gestation were fed one time with: VPA, 800 mg/kg b.w. or THAL, 500 mg/kg b.w. VPA was mixed with 1 ml of saline solution and THAL was mixed with vegetables oil and both were administrated orally. Control animals were fed with 1 ml of mixture of oil and saline, 1:1 v/v (Kolozsi et al., 2009; Narita et al., 2010). Figure 1 shows a schematic diagram of all the procedures that each animal has undergone.

For each test group in our study the animals came from two litters. At the onset of our experiments we started with 40 rat pups. Out of the initial number, 6 pups died during the second narcosis accompanying the microdialysis experiments and were excluded from further analysis: three pups (1F - female and 2M - male) from the control group, two (2F) from the VPA-treated group and one (1M) from the THAL-treated group. In the final analysis, there were 10 (6F + 4M) control animals, 11 (5F + 6M) VPA-treated ones and 13 (4F + 9M) THAL-treated ones.

### Behavioral Test: Ultrasonic Vocalizations (USV).

On PND 4-5, pups’ paws were marked with Ketchum animal tattoo ink (Ketchum Manufacturing INC., Canada) inserted subcutaneously through a 30-gauge hypodermic needle. The number of ultrasonic vocalizations (USV) was determined in all infant rats at PND 9. At that age, rat weight of all subjects in the group expressed as a mean value ± SD was 16 ± 3 g for control, for VPA group – 19 ± 3 g and for THAL group – 20 ± 2 g. Pups’ USV were evoked by isolation from the dam and littermates. First, the entire litter was removed from the home cage and placed in a waiting chamber on the home cage bedding on a heating pad, set at 35°C in a Styrofoam box (24 × 27.5 × 21 cm) for at least 10 min. Pups were then taken individually from the box, placed in a glass container with fresh bedding, and lowered inside a Styrofoam box (17 × 17 × 17 cm) with CM16/CMPA ultrasound condenser microphone (Avisoft Bioacoustics, Germany) located in the lid of the box. The microphone was connected *via* UltraSoundGate 116Hb device (Avisoft Bioacoustics, Germany) to a personal computer. The audio tracks were monitored in real time and recorded using Avisoft Recorder USGH (Avisoft Bioacoustics, Germany) and registered to a.wav file with a sampling rate of 250 kHz in a 16-bit format. The recording session lasted 5 min. After the session, the pups were weighted and returned to the waiting chamber. After the whole litter was tested, the animals were returned to their home cage. Collected data were analyzed using SASLab Pro (Avisoft Bioacoustics, Germany) in a two stage manner (i.e., automatic and manual detection) using a fast Fourier transform (512 FFT-length, 100% frame, FlatTop window and 75% temporal resolution overlap) with a high-pass cut-off frequency of 20 kHz, which removed non-ultrasounds from further analysis. Spectrograms were generated at 488 Hz frequency resolution and 0.512 ms time resolution. In order to enhance the automatic detection of sounds, linear predictive coding was used at 30 coefficients, which reduced noise on the spectrogram. The software automatically detected sounds using a power threshold of -52 dB and detection hold time of 40 ms. The resulting spectrogram with highlighted sounds was then manually reviewed and corrected if necessary by an experienced user. The representative spectrograms of USV obtained from each group were presented in the Figure 4C

### Magnetic Resonance Spectroscopy

Magnetic resonance spectroscopy was performed using 7 T Bruker BioSpec 70/30 Avance III system. Animals 30 (±2) day old were anesthetized with 1.5–2% isoflurane in oxygen and head-first prone positioned in the MR compatible animal bed. At that age the rat weight of F subgroup /M subgroup/all subjects in the group expressed as a mean value ± SD was 60 ± 15 g/65 ± 10 g/63 ± 13 g for control, for VPA group – 58 ± 5 g/61 ± 6 g/60 ± 5 g and for THAL group – 60 ± 10 g/63 ± 8 g/61 ± 10 g. Transmit cylindrical radiofrequency coil (8.6 cm inner diameter) and a rat brain-dedicated receive-only array surface coil (2 × 2 elements) were positioned over the animal’s head. Respiration rate and body temperature were monitored throughout the experiment with a small animal monitoring system. Positioning tripilot scans were performed, followed by high resolution T2-weighted TurboRARE structural scan (TR/TEeff = 2500/33 ms, RARE factor = 8, spatial resolution = (117 × 117 × 700 μm), FOV = 30 × 30 mm, 22 slices, 400 μm gap, NEX = 2, Scan Time = 3 min) covering the entire brain. Using the high-resolution structural image, volume of interest (VOI) encompassing hippocampus was planned for MRS acquisition with the VOI dimension: 7 × 1.5 × 2 mm. Prior to spectrum acquisition, an extensive shimming procedure was performed in order to maintain an unsuppressed water line width (FWHM) below 15 Hz, covering linear and second order global shims, followed by linear and second order local shimming with the FASTMAP protocol within the measured VOI. The shimming procedure lasted 10–15 min. Subsequently, the spectrum was acquired with short echo time PRESS sequence (TR/TE = 2500/20 ms, 512 averages, 2048 points, scan time = 17 min) with VAPOR water suppression, the outer volume suppression, frequency drift correction (flip angle – 5°) and eddy current correction.

**FIGURE 2:**
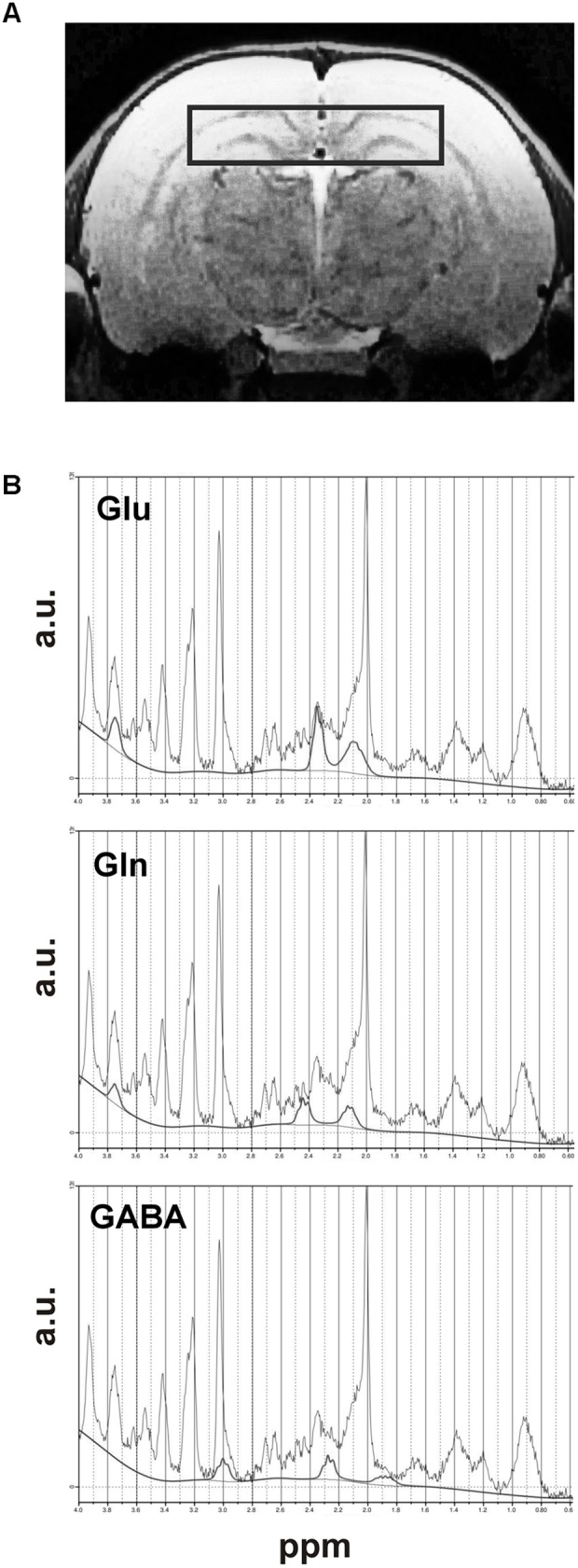
Dorsal hippocampus: **(A)** MRI scan showing VOI of brain used for MRS studies and **(B)** the graphs presenting an example of MRS spectra of Glu, Gln, and GABA signals with fitted lines.

Localization of volume of hippocampus used for MRS measurements was presented in Figure 2A. Metabolite concentrations were estimated using the LCModel software (Provencher, 1993) in a fully automated pipeline. Absolute metabolite concentrations were determined with reference to unsuppressed water, uncorrected for relaxation. Water scaling was performed by the same software: each metabolite peak was normalized by expressing its magnitude as a ratio of the magnitudes of metabolite and the unsuppressed water peak. The representative MRS spectra with Glu, Gln and GABA lines as fitted by the program were presented in Figure 2B.

### *In vivo* Microdialysis in Anesthetized Rats

The 31 (±2)-day-old Wistar rats of both sexes were used for microdialysis experiments. One hour after urethane anesthesia (1.25 g/kg intraperitoneally) the rats were placed in a stereotactic frame (Stoelting, United States). The rat skin was cut in the middle of the head, a hole was drilled in the skull cover, and the microdialysis probe (CMA/11 14/01 Cupr) was stereotactically implanted into the right hippocampus [coordinates: anteroposterior (AP), -3.5 mm; mediolateral (ML), -1.4 mm; dorsoventrally (DV), -3 mm (Paxinos and Watson, 1982)]. Artificial cerebrospinal fluid (aCSF; NaCl 122 mM, KCl 3 mM, MgSO_4_ × 7H_2_O 1.2mM, KH_2_PO_4_ 0.4 mM, NaHCO_3_ 25 mM, CaCl_2_ × 2H_2_O 1.2 mM, HEPES 4 mM, pH = 7.4) at room temperature (RT) was pumped through the probe at a flow rate of 2 μl/min. We started to collect microdialysates samples 30 min after implanting the probe and samples were collected every 40 min during 4 h. The collected samples were stored at -70°C until we were able to perform HPLC tests. After completing microdialysis, the animals were decapitated and the correct localization of the probes in the right hippocampus was verified. The inspection showed that all microdialysis probes were correctly implanted. The left (intact) hippocampi were dissected, placed in 500 μl of PBS, gently homogenized with teflon homogenizer and then processed as described below in Sections “High-Performance Liquid Chromatography (HPLC) Analysis of Amino Acids” and “Sample Preparation.”

### High-Performance Liquid Chromatography (HPLC) Analysis of Amino Acids

High-performance liquid chromatography is a technique of analytical chemistry useful for separation, identification and determination of compounds that are dissolved in trace concentrations as low as nano- and even picomolar. A reversed-phase HPLC with precolumn derivatization of primary amino acids with *o*-phthaldialdehyde is a method of choice for the determination of amino acids concentrations.

#### Homogenates

In order to extract the free amino acids, frozen 100 μl aliquots of homogenates were thawed, mixed with 9 volumes of acidified methanol (8.4 ml 0.1 M HCl/100 ml methanol). The mixture was left for 20 min at 4°C and centrifuged for 10 min at 15 400 × *g*. The supernatant fraction was then maintained at -70°C until determination of free amino acids by HPLC (Francis and Lowe, 1993), see below.

#### Microdialysates

**FIGURE 3:**
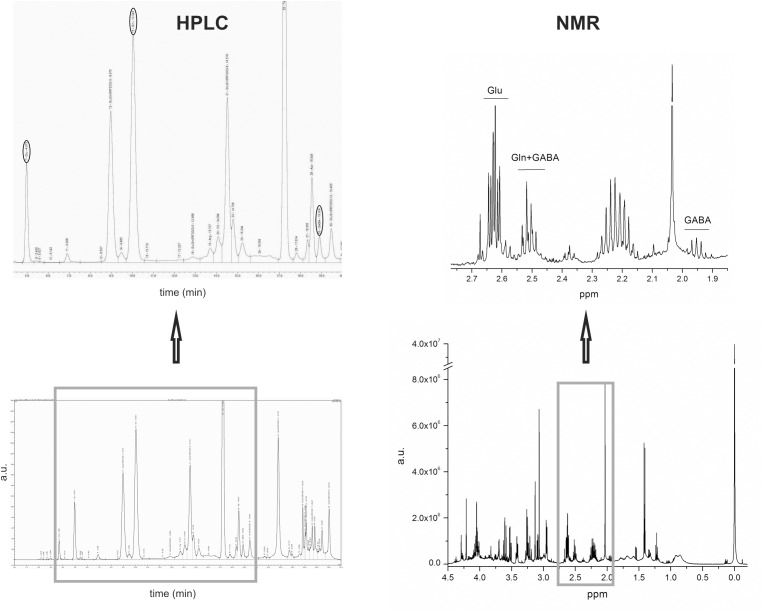
The example of brain extract HPLC chromatogram with Glu, Gln, and GABA peaks marked with ovals is on the left hand-side of the figure and the example of brain extract NMR spectra with indicated signals from Glu, Gln, and GABA is on the right hand-side of the figure.

Amino acids were assayed in microdialysate samples using HPLC with fluorescence detection after pre-column derivatization in a timed reaction with *o*-phtalaldehyde plus mercaptoethanol, as described by Kilpatrick (1991). Derivatized samples (25 μl) were injected on to a 250 × 4.6 mm 5 μm Hypersil Gold BDS C18 column with a mobile phase of 50 mM phosphate buffer (KH_2_PO_4_/K_2_HPO_4_) containing 10% v/v methanol, pH 6.2 (solvent A), and methanol (solvent B). The representative chromatogram with marked (in ovals) peaks of Glu, Gln and GABA is presented in Figure 3.

### Nuclear Magnetic Resonance Spectroscopy

Nuclear magnetic resonance spectroscopy allows to obtain information about many metabolites in biological fluid sample in a single measurement. This method is relevant only for measuring metabolites with low molecular weight (<1000 Da) with the exception of sphingolipids for which the maximum molecular weight is 1500 Da (Wishart, 2008). Compared to other analytical techniques, NMR spectroscopy has special characteristics, which make it uniquely suitable for the analysis of metabolite mixtures. NMR allows reliable detection and quantification of a wide range of metabolites containing hydrogen, present in complex biological fluids at micromolar concentrations. NMR is considered to be a non-destructive technique with low handling and preprocessing times.

#### Sample Preparation

Fresh 400 μl aliquots of hippocampal homogenates were extracted for NMR studies according to the procedure by Bligh and Dyer (1959) with slight modification, the homogenates were vortexed for 1 min with 1875 μl mixture of 99% methanol, 98% chloroform and 36% HCl, 40:20:1 (v/v). As the next step, 625 μl chloroform was added and the mixture was again vortexed for 1 min. After that, 625 μl of water was added and vortexed for 1 min. Then the mixture was centrifuged at 2000 × *g* for 15 min using swing out rotor to obtain three phases: upper – water/methanol containing amino acids and other substances diluted in water, lower – containing lipids and middle – containing proteins. Upper and lower phases were extracted for NMR examination. The middle phases were collected in order to evaluate total protein concentrations in the samples (using Lowry test). This data was used to normalize the results of amino acid levels in hippocampus, using both: NMR and HPLC methods. Before measuring levels of amino acids using NMR spectrometer, the water/methanol phase of the sample was dried using nitrogen. Dry residues were then diluted in 700 μl of D_2_O and immediately tested.

#### Spectra Acquisition

The pH of samples was stabilized at 7.5 ± 0.2 using HCl. Three-trimethylsilyl propionic acid (TSP) at 1 mM final concentration in the sample was used as an internal reference for normalization of all spectra and quantitative statistical analysis. All NMR spectra were acquired at 25°C using Avance III HD 500MHz spectrometer (Bruker, Germany). Excitation sculpting (Hwang and Shaka, 1995) was used to suppress the water signal while minimizing phase distortion of the spectrum and utilized a 2 ms square inversion pulse in a double pulse field gradient spin echo. Line broadening of 0.5, baseline and phase correction were applied to each spectrum using software implemented in spectrometer.

Concentrations of Glu, Gln, and GABA were calculated from the spectra and used for further statistical analysis. Glu was quantified using the (^3^CH_2_) at 2.61 ppm and Gln using the multiplet at 2.51 ppm (^4^CH_2_) overlapped with GABA triplet (^2^CH_2_). GABA was quantified using the isolated quintet at 1.95 ppm (^3^CH_2_). Figure 3 presents the representative NMR spectra of Glu, Gln, and GABA. Portion of the obtained results, including the concentrations of Glu, Gln, and GABA was used in this publication, while the remainder will be used in subsequent metabolomic studies.

### Statistical Analysis

Univariate statistical analysis was done using SPSS Statistics software package (IBM Company, United States), which was performed for all data using one-way ANOVA test followed by Dunn’s correction. P values lower than 0.05 were considered as significant. For the outlier detection we used a test provided by the same software.

Multivariate statistical analysis was performed using supervised methods of orthogonal partial least square discriminant analysis (OPLS-DA). Mean-centering and unit variance scaling were performed before analysis. In the OPLS-DA modeling the goodness of fit is reported as the cumulative scores across all of the components: R2cum – explained by the model and Q2cum – predicted by the model. OPLS-DA model was considered significant if R2cum and Q2cum were significantly larger than zero and was considered as good when both values were equal or greater than 0.5 (Bylesjo et al., 2008). The variable importance in the projection (VIP) value of each variable in the model was calculated to indicate its contribution to the classification. Those variables with VIP value greater than 1.0 were considered significantly different, and a larger VIP value of a variable represented higher contribution to the discrimination between groups (Karp et al., 2005; Toczylowska et al., 2013). We validated the model by applying CV-ANOVA (Analysis of Variance testing of Cross-Validated predictive residuals) test using Jack-knifing uncertainties. Strong outliers were removed only for building the OPLS-DA model. They can have high impact on the model, shifting it significantly and reducing its predictability and are identified from the scores and Hotelling’s *T*^2^ range plots. The latter is a multivariate generalization of Student’s *t*-test, providing a check for the level of observation adherence to multivariate normality, when used in conjunction with a scores plot, the Hotelling’s *T*^2^ defines the normality area corresponding to 95% confidence. Further analysis of the model was performed on all subjects in the groups (without removing any of them as outliers). Multivariate analysis, OPLS-DA, were performed using SIMCA software package, ver. 14, MKS Umetrics, Sweden (Ellis et al., 2007).

## Results

### Evaluation of Autism-Like Behavior: Ultrasonic Vocalization

**FIGURE 4:**
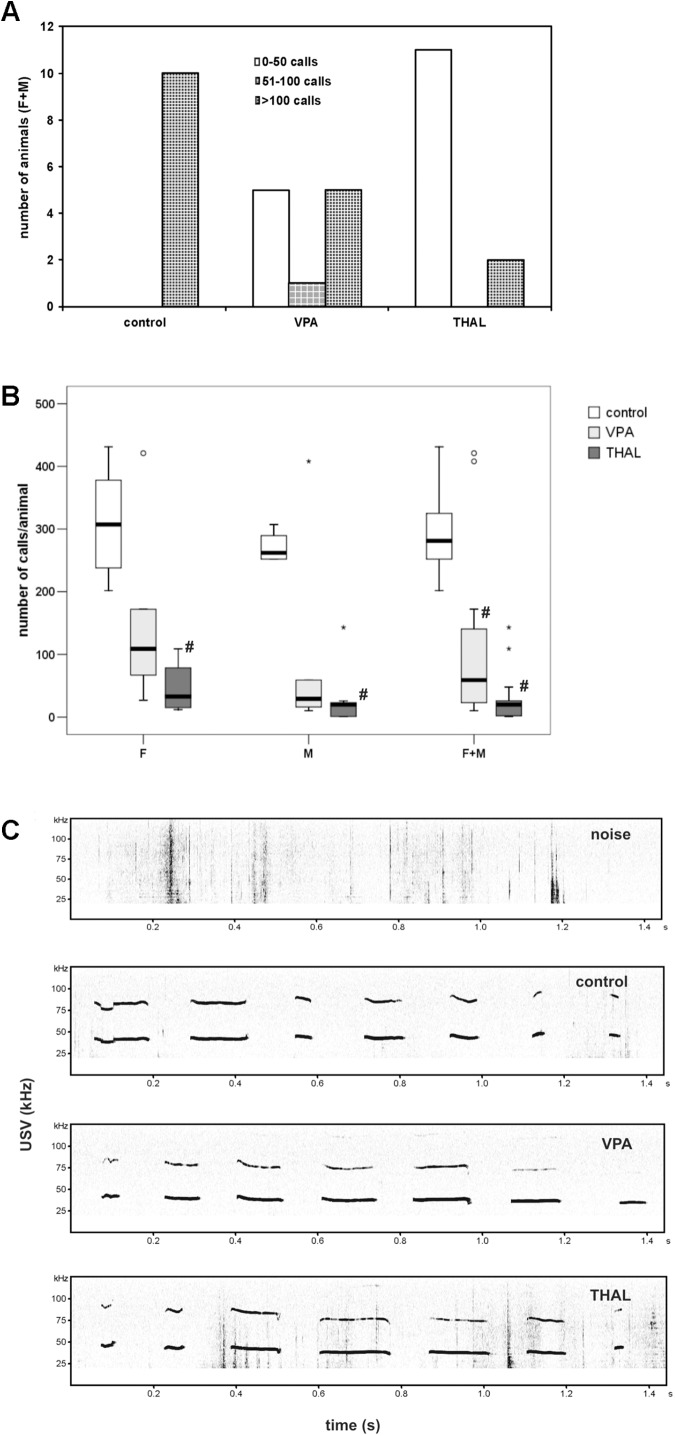
**(A)** The number of vocalizing animals for each study (control, VPA, THAL) group, each separated into three subgroups according to the number of calls emitted; subgroup 1: 0–50 calls; subgroup 2: 51–100; subgroup 3: above 100. VPA, rat pups in group treated with valproic acid; THAL, treated with thalidomide, all treated during fetal development; F, female; M, male animals. **(B)** Box and Whiskers plot of number of calls per animal obtained from ultrasonic vocalization test expressed as a median (min-max) value. Number of rat pups: *n* = 10 (control), *n* = 11 (VPA) and *n* = 13 (THAL); # represents results statistically significant vs. control, *p* < 0.05, one-way ANOVA followed by Dunn’s correction; box containing 50% of results that fell in the range between 25% (bottom line) and 75% (top line), (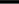) median value, 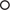 – outlier point per rat, ^∗^ – extreme outlier point per rat. **(C)** The example of spectrograms of USV from each group.

Number of USV is presented in Figure 4. The animals were divided into three groups based on number of calls: first was 0–50 calls, second 51–100 and third more than 100 calls during the time of measurement. All control animals belong to the third group. VPA animals were mostly in the first or the third group (41% in each group), while only 18% were in the second group. Among the THAL rats, 85% of the animals were in the first group and 15% in the third one (Figure 4A). Figure 4B presents number of calls per animal as median (min-max value and percentiles: 75/85/95) in each of the three subgroups designated by gender (F and M) and both (F + M). In the control group, 281 (202–431, percentiles: 325/378/431) calls was observed in the F + M subgroup, 308 (202–431, percentiles: 378/431/431) calls in the F subgroup and 262 (252–307, percentiles: 290/307/307) calls in the M subgroup. The THAL group was the most different from the control group, in which the number of calls decreased in F + M, F and M subgroups to levels of 20 (1–143, percentiles: 26/109/143), 33 (12–109, percentiles: 79/109/109) and 20 (1–143, percentiles: 23/26/143), respectively. In the VPA group, statistically significant differences from the control were demonstrated only in the F + M subgroup in which the number of calls decreased to 59 (10–421, percentiles: 172/408/421). There were no statistically significant differences in calls between subgroups of the VPA and THAL groups.

### MRS *in vivo* Measurements of Glu, Gln, and GABA Content in Rat Hippocampus

**FIGURE 5:**
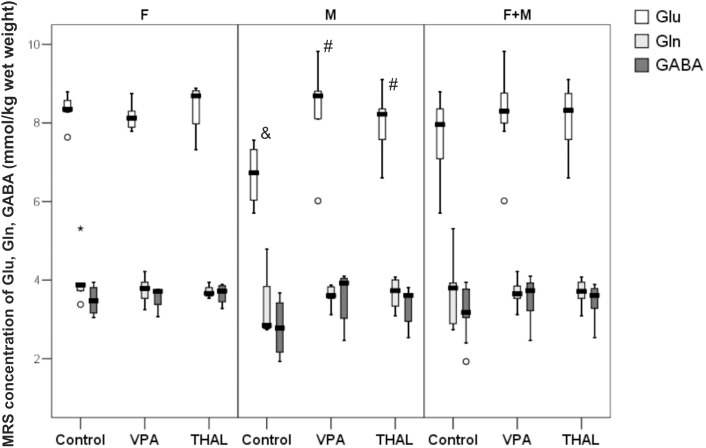
Box and Whiskers plot of changes in Glu, Gln, and GABA levels in rat hippocampus measured in vivo using MRS method. The obtained results are expressed in mM/kg wet weight). Results that are statistically significant vs. control are represented by # and within a group between genders by &, *p* < 0.05. Number of rat pups: *n* = 10 (control), *n* = 11 (VPA), and *n* = 13 (THAL); # represents results statistically significant vs. control, *p* < 0.05, one-way ANOVA followed by Dunn’s correction; box containing 50% of results that fell in the range between 25% (bottom line) and 75% (top line), (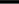) median value, 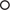 – outlier point per rat, ^∗^ – extreme outlier point per rat.

Figure 5 presents concentrations of Glu, Gln, and GABA measured *in vivo* in rat hippocampus using the MRS method. The obtained results are expressed in mmol/kg wet weight. Statistically significant differences between the control and test groups were observed exclusively of Glu content in male (M) subgroups. We observed Glu concentration increase of 20% in the VPA group and 18% in the THAL group. For control M subgroup, Glu concentration median (min-max and percentiles: 75/85/95) value was at the level of 6.73 (5.70–7.56, percentiles: 7.32/7.56/7.56) while in VPA and THAL groups these values were 8.46 (6.02–8.81, percentiles: 8.81/9.82/9.82) and 8.22 (6.60–9.10, percentiles: 8.36/8.93/9.10), respectively. We have observed trend of increased GABA levels for each gender subgroup. The difference was smallest for the control group and greater for VPA and greatest for the THAL group, however, the differences between the groups were not statistically significant. Notably, we also noticed a 21% difference in median values of Glu levels between genders in the control animals which were 6.73 (5.70–7.56, percentiles: 7.32/7.56/7.56) and 8.35 (8.28–9.45, percentiles: 8.57/8.79/8.79) for M and F subgroups, respectively.

### HPLC Analysis of Microdialysates Based on *in vivo* Measurements of Extracellular Concentrations of Glu, Gln and GABA in Rat Hippocampus

**FIGURE 6:**
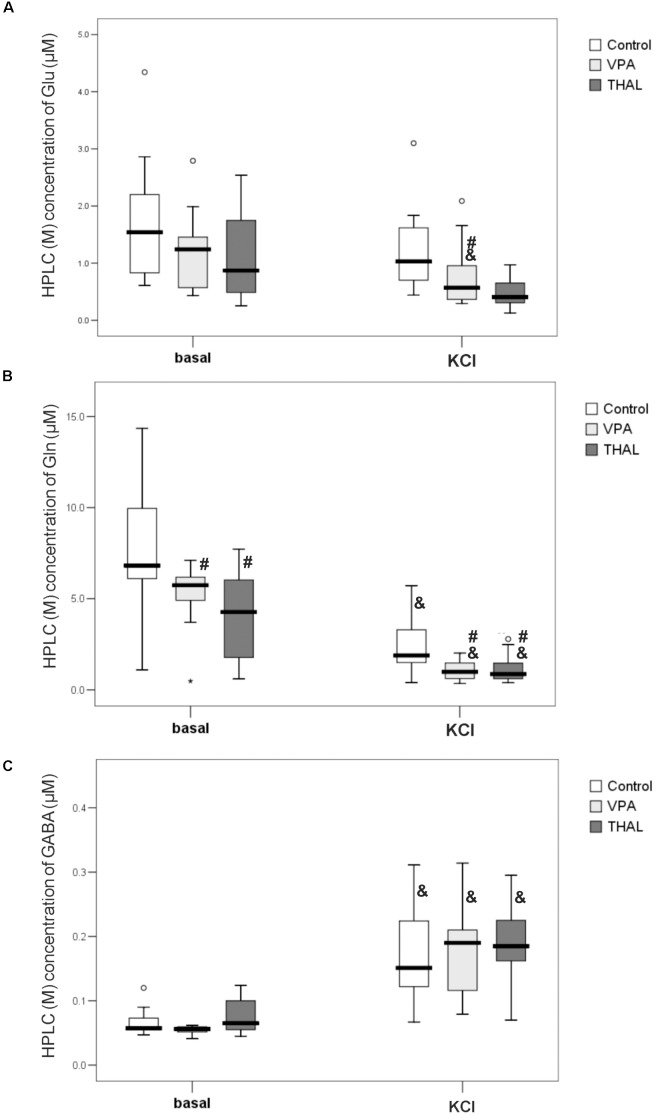
Box and Whiskers plot of concentration of Glu **(A)**, Gln **(B)** and GABA **(C)** in microdialysates of rat hippocampus collected at basal level and at 40 min post application of 100 mM KCl (KCl) measured using HPLC method. Results are presented as median (min÷max) values of tested amino acid concentration in μM. Results that are statistically significant vs. control represented by # and vs. basal level by &, *p* < 0.05. Number of rat pups: *n* = 10 (control), *n* = 11 (VPA) and *n* = 13 (THAL); # represents results statistically significant vs. control, *p* < 0.05, one-way ANOVA followed by Dunn’s correction; box containing 50% of results that fell in the range between 25% (bottom line) and 75% (top line), (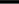) median value, 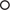 – outlier point per rat, ^∗^ – extreme outlier point per rat.

Figures 6A–C shows the results of HPLC analysis of microdialyzate samples collected during two 40-min periods, representing μM concentrations of Glu (A), Gln (B), and GABA (C), measured before (basal level) and after stimulating hippocampus with 100 mM KCl (KCl). For this measurement method, we did not partition the samples into gender based subgroups. In our study we measured the basal levels of Glu, Gln, and GABA median (min–max and percentiles: 75/85/95) values in the control group to be 1.54 (0.61–4.34, percentiles: 2.20/2.86/4.34), 6.83 (1.09–14.35, percentiles: 9.96/13.82/14.35), and 0.06 (0.05–0.12, percentiles: 0.09/0.12/0.12), respectively. As presented in Figures 6A,C in VPA and THAL groups, median of basal levels of Glu and GABA did not differ from control. For Gln concentrations (Figure 6B), the median values for autistic-like animals were significantly lower than control: in VPA by 16% and in THAL groups by 37%. For the control group, the median value of basal level of Gln was 6.83 (1.09–14.35, percentiles: 9.96/13.82/14.35), for VPA 5.74 (0.48–7.12, percentiles: 6.22/6.40/7.12) and for THAL group it was 4.27 (0.60–7.73, percentiles: 6.03/6.46/7.73). For all the experimental groups, KCl stimulation resulted in a significant increase in GABA concentration in microdialysates compared to corresponding basal levels, the concentration for control group increased by 150%, for VPA by 217% and for THAL by 171%. The median values for the control group increased from 0.06 (0.05–0.12, percentiles: 0.09/0.12/0.12) to 0.15 (0.07–0.31, percentiles: 0.22/0.23/0.31), for VPA from 0.06 (0.04–0.06, percentiles: 0.06/0.06/0.06) to 0.19 (0.08–0.31, percentiles: 0.21/0.28/0.31) and for THAL increased from 0.07 (0.05–0.12, percentiles: 0.10/0.11/0.12) to 0.19 (0.07–0.29, percentiles: 0.23/0.26/0.30). The above data shows no significant differences in post stimulation results between study groups (Figure 6C). Gln concentration in samples of dialysates collected after KCl stimulation decreased significantly as compared to the corresponding basal levels in all groups (Figure 6B), for the control group by 72%, for VPA group by 93% and for THAL group by 80%. The median values for the control group decreased from 6.83 (1.09–14.35, percentiles: 9.96/13.82/14.35) to 1.89 (0.40–5.72, percentiles: 3.30/4.15/5.72), for VPA from 5.74 (0.48–7.12, percentiles: 6.22/6.40/7.12) to 0.99 (0.35–2.02, percentiles:1.52/1.64/2.02) and for the THAL group from 4.27 (0.60–7.73, percentiles: 6.03/6.46/7.73) to 0.87 (0.39–2.79, percentiles: 1.47/2.48/2.79). These decreases were significantly more pronounced in both experimental groups than in control (Figure 6B). KCl stimulation also induced a decrease of dialysates Glu concentrations in experimental groups as compared to the control group and post stimulations levels vs. corresponding basal levels for all groups. These changes were statistically significant only in the VPA group; the Glu value decreased by 54% as compared to the basal level and the median value decreased from 1.24 (0.44–2.80, percentiles: 1.53/1.99/2.80) to 0.57 (0.30–2.09, percentiles: 1.05/1.66/2.09) which was 45% lower than the control which was 1.03 (0.44–3.10, percentiles: 1.62/1.83/3.10).

### Total Glu, Gln, and GABA Concentrations in Rat Hippocampus Homogenates: *Ex vivo* Measurements Using HPLC Method

**FIGURE 7:**
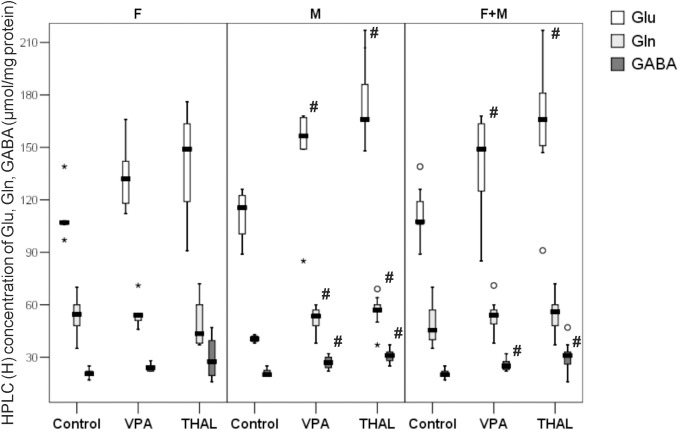
Box and Whiskers plot of total Glu, Gln, and GABA concentration in rat hippocampus homogenates measured using HPLC method. Results are presented as median (min-max) values of tested amino acids concentration. Differences statistically significant vs. control are represented by # *p* < 0.05. Number of rat pups: *n* = 10 (control), *n* = 11 (VPA) and *n* = 13 (THAL); # represents results statistically significant vs. control, *p* < 0.05, one-way ANOVA followed by Dunn’s correction; box containing 50% of results that fell in the range between 25% (bottom line) and 75% (top line), (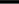) median value, 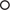 – outlier point per rat, ^∗^ – extreme outlier point per rat.

The concentrations of amino acids are expressed in μmol per mg of protein and are presented in Figure 7. In both VPA and THAL groups vs. the control, we observed statistically significant increases in concentrations of all tested compounds only in male animals (M subgroup). The level of Glu increased in the VPA group by 41%, Gln by 39% and GABA level increased by 40%. The Glu median (min-max and percentiles: 75/85/95) levels increased from 116 (89–126, percentiles: 123/126/126) to 164 (149–168, percentiles: 167/168/168), Gln from 41 (38–43, percentiles: 42/43/43) to 57 (48–71, percentiles: 60/64/69) and GABA level increased from 20 (19–25, percentiles: 23/25/25) to 28 (24–32, percentiles: 30/32/32). In the THAL group, Glu concentration increased by 43%, Gln by 39% and GABA concentration increased by 55%. In the THAL group, Glu concentration median (min-max and percentiles: 75/85/95) values increased from 116 (89–126, percentiles: 123/126/126) to 166 (148–217, percentiles: 186/207/217), Gln concentration increased from 41(38–43, percentiles: 42/43/43) to 57 (37–69, percentiles: 60/64/69) and GABA concentration increased from 20 (19–25, percentiles: 23/25/25) to 31 (25–37, percentiles: 33/33/37). Gender specific differences were also observed in concentrations of Gln between the control group and both, VPA and THAL groups. In male animals, the median value of Gln level increased by about 40%, in females it decreased by about 10% in the VPA group and by 20% in the THAL group. In F + M subgroups of VPA and THAL groups, only Glu and GABA concentrations were significantly elevated. Median value of Glu content in VPA group increased by 38%, from 108 (89–139, percentiles: 119/126/139) to 149 (85–168, percentiles: 166/167/168) and GABA content increased by 25%, from 20 (17–25, percentiles: 22/25/25) to 25 (22–32, percentiles: 28/30/32). In the THAL group, median value of Glu increased by 54%, from 108 (89–139, percentiles: 119/126/139) to 166 (91–217, percentiles: 181/207/217) and median value of GABA increased by 55% from 20 (17–25, percentiles: 22/25/25) to 31 (16–47, percentiles: 33/37/47).

### *Ex vivo* Measurements of Total Glu, Gln, and GABA Concentrations in Rat Hippocampus Homogenates: NMR Examinations

**FIGURE 8:**
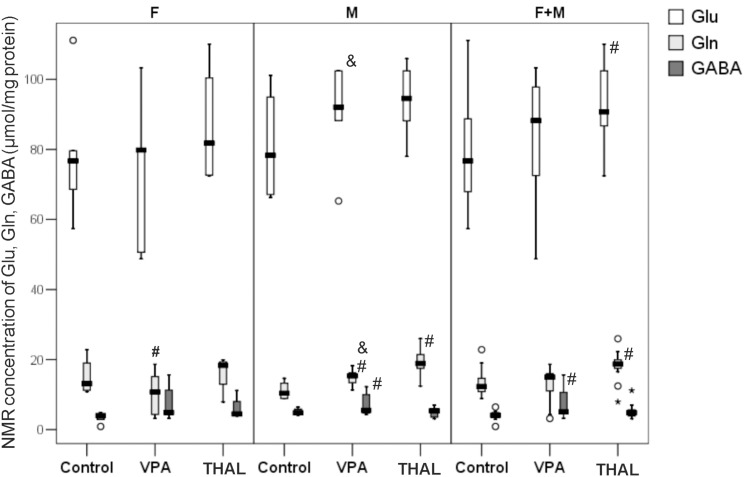
Box and Whiskers plot of total Glu, Gln, and GABA concentrations of rat hippocampus homogenates measured using NMR method. Results are presented as median (min-max) values of tested amino acids level. Differences statistically significant vs. control are represented by # and within a group between genders by &, *p* < 0.05. Number of rat pups: *n* = 10 (control), *n* = 11 (VPA), and *n* = 13 (THAL); # represents results statistically significant vs. control, *p* < 0.05, one-way ANOVA followed by Dunn’s correction; box containing 50% of results that fell in the range between 25% (bottom line) and 75% (top line), (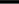) median value, 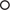 – outlier point per rat, ^∗^ – extreme outlier point per rat.

The amino acids concentrations presented in Figure 8 are expressed in μmol per mg of protein. The statistically significant differences in concentrations of Gln vs. control were detected in male animals of both, VPA and THAL groups and in females only in the VPA group. We observed differences between gender subgroups in the VPA group and the control group; in males, the concentration of Gln increased by 60% and in females it decreased by 15%. In the comparison of VPA group and the control group, the median (min-max and percentiles: 75/85/95) values of Gln concentration in male animals increased from 10 (9–15, percentiles: 13/15/15) to 16 (13–19, percentiles: 16/18/18) and in females it decreased from 13 (11–23, percentiles: 19/23/23) to 11 (3–19, percentiles: 15/19/19). Differences in Glu concentrations were also observed between genders: in females the median concentration values of this amino acid had not changed and in males it increased by 18%, from 78 (66–101, percentiles: 95/101/101) to 92 (88–103, percentiles: 102/103/103). In the THAL group vs. the control group, Gln concentration increased by 90%, from10 (9–15, percentiles: 13/15/15) to 19 (12–26, percentiles: 21/22/26) only in male rats. We observed statistically significant increase of GABA concentration by 20%, from 5 (4–6, percentiles: 6/6/6) to 6 (4–12, percentiles: 10/12/12) in the male VPA group as compared to the control. In the THAL group, we observed an increase of Glu concentration by 18%, from 77 (57–111, percentiles: 89/101/111) to 91 (72–110, percentiles: 102/106/110) and increase of Gln concentration by 58%, from 12 (9–23, percentiles: 15/19/23) to 19 (8–26, percentiles: 20/22/26) for both F and M subgroups.

### Discriminant Analysis

OPLS-DA analysis of the amino acid concentrations allows to build models from HPLC: microdialysis and total homogenate, and NMR data. For MRS data no model could be built. All of the statistical analyses for HPLC (microdialysis) samples were performed only on the differences between basal values and values post KCl stimulation. Strong outliers were removed only for model building, however, all samples in the groups were used for further analysis. R2cum results from OPLS-DA analysis were also presented separately as a part that was correlated (predictive) to groups and as a part that was uncorrelated (orthogonal) to groups. For models there was no orthogonal component in the data (PCA).

**FIGURE 9:**
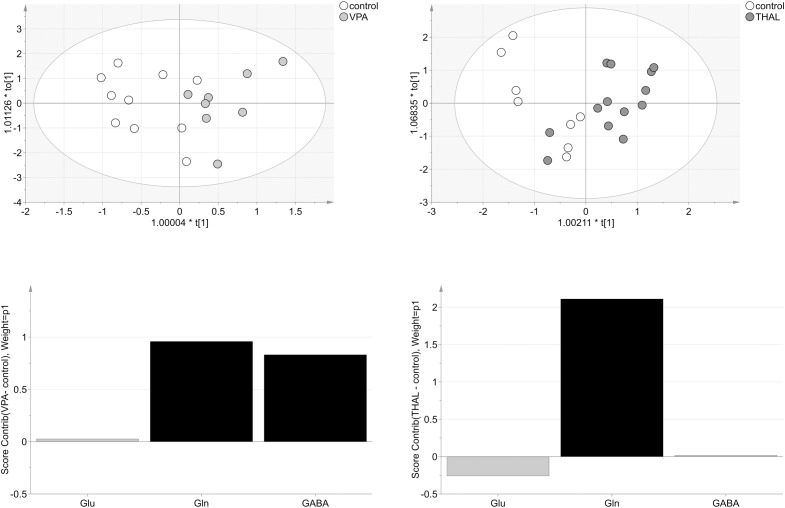
The score plots of the two-component OPLS-DA model for HPLC (M) data for VPA and THAL vs. control group; t_o_[1] represents variation within class for the first orthogonal component, whereas t[1] represents variation between classes for the first predictive component. Ellipse represents Hotelling T2 with 95% confidence. Lower graphs are the score contribution of differences of VPA vs. control and THAL vs. control. Black is assigned to compounds with VIP > 1. Positive value means greater concentration than control and negative means lower.

For HPLC (microdialysis) analysis of VPA vs. control group, three VPA samples were removed as strong outliers before model building. The model consisted of one predictive and one orthogonal component (Figure 9). For this model, R2cum and Q2cum were 65% (15% - predictive and 50% - orthogonal) and 52%, respectively. Moreover, the model correctly classified 81% of the analyzed samples to appropriate groups (*p* = 0.006). The model was validated using CV-ANOVA, *p* = 0.04. The most important amino acids (VIP > 1) involved in group differentiation were Gln and GABA. For THAL vs. control group, the model consisted of one predictive and one orthogonal component (Figure 9). During model building two control samples were removed as strong outliers. The resulting model described 67% (29% – predictive and 38% – orthogonal) of the data (R2cum) and predicted 46% of the data (Q2cum). The model correctly classified 74% of the samples to the appropriate groups (*p* = 0.04) and was validated using CV-ANOVA, *p* = 0.04. The most important amino acid (VIP > 1) used for group differentiation was Gln. From the analysis of the experimental groups vs. control we found moderate positive correlation of both Gln and GABA for VPA group but only Gln for the THAL group.

**FIGURE 10:**
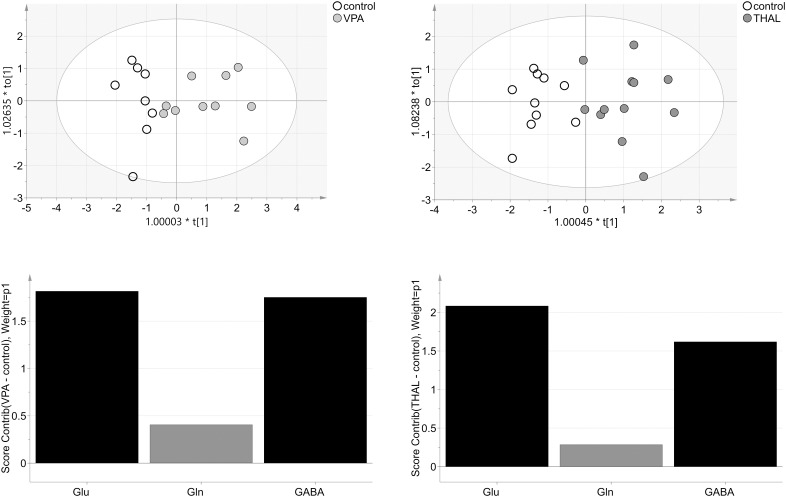
The score plots of the two-component OPLS-DA model for HPLC (H) data for VPA and THAL vs. control group; t_o_[1] represents variation within class for the first orthogonal component, whereas t[1] represents variation between classes for the first predictive component. Ellipse represents Hotelling T2 with 95% confidence. Lower graphs are the score contribution of differences of VPA vs. control and THAL vs. control. Black is assigned to compounds with VIP > 1. Positive value means greater concentration than control and negative means lower.

OPLS-DA model from HPLC (homogenate) studies of VPA vs. control group consisted of one predictive and one orthogonal component (Figure 10). For model building, two controls and one VPA data samples were removed as strong outliers. The obtained model described 96% (68% – predictive and 28% – orthogonal) (R2cum) of the data and predicted 58% (Q2cum) of the data. As much as 90% of the samples were correctly classified to appropriate groups (*p* = 0.001): 100% control samples and 80% VPA samples from which two of the ten were erroneously classified as control samples. Model was validated using CV-ANOVA, *p* = 0.02. Model for THAL vs. control group consisted of one predictive and one orthogonal component as well (Figure 10). Before model building, one THAL data sample was removed as strong outlier. Obtained model described 91% of the data (60% – predictive and 31%- orthogonal) (R2cum) and predicted 75% of the data (Q2cum). In this model 100% of the samples were correctly classified (*p* < 0.0001) to appropriate groups, the model was validated using CV-ANOVA, *p* = 0.0001. We observed that the model obtained from the THAL group data performed better separation of the samples than the model obtained using the VPA group. Glu and GABA were the most significant (VIP > 1) amino acids involved in group differentiation for both models (Figure 10). The analysis of experimental groups vs. control indicated that for both groups, the Glu and GABA levels had strong positive correlation while Gln had weak positive correlation.

**FIGURE 11:**
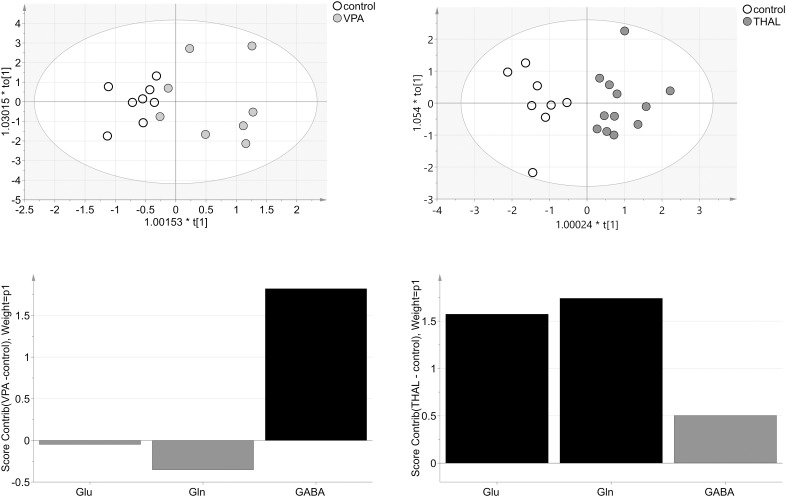
The score plots of the two-component OPLS-DA model for NMR data for VPA and THAL vs. control group; t_o_[1] represents variation within class for the first orthogonal component, whereas t[1] represents variation between classes for the first predictive component. Ellipse represents Hotelling T2 with 95% confidence. Lower graphs are the score contribution of differences of VPA vs. control and THAL vs. control. Black is assigned to compounds with VIP > 1. Positive value means greater concentration than control and negative means lower.

OPLS-DA model for VPA vs. control group obtained from NMR data consisted of one predictive and one orthogonal component (Figure 11). For model building, two control and three VPA data samples were removed as strong outliers. For the resulting model, R2cum was 95% (67% – predictive and 28% – orthogonal) and Q2cum was 57%. In this model, 88% of the samples were correctly classified (*p* = 0.004) to appropriate groups. The model was validated using CV-ANOVA, *p* = 0.04. The model for THAL vs. control group also consisted of one predictive and one orthogonal component (Figure 11). For model building, two controls and one THAL data samples were removed as strong outliers. The resulting model fit 80% of the data (60% – predictive and 31% – orthogonal) (R2cum) and was able to predict 77% of the data (Q2cum). This model correctly classified 100% of the samples (*p* = 0.0001) to appropriate groups and was validated using CV-ANOVA, *p* = 0.0001. In the VPA model, the most important amino acid (VIP > 1) involved in group differentiation was GABA while in the THAL model both Glu and Gln. The analysis of experimental groups vs. control indicated that both Gln and Glu had moderate positive correlation for the THAL group while for the VPA group correlation was weak negative. GABA had positive correlation for both groups: THAL (moderate) and VPA (strong). We observed that the model from NMR data for the THAL group better differentiated the samples than the model for VPA group.

Mean sensitivity of the methods for both models (VPA vs. control and THAL vs. control) was 82.15% for NMR and 81.82% for HPLC. Mean specificity for NMR and HPLC was 85.71% and 90%, respectively. Sensitivity and specificity were lower for VPA vs. control group than for THAL vs. control group. Concluding from these results, group differentiation based on hippocampus neurotransmitters analysis can obtained using either NMR spectroscopy or HPLC method.

## Discussion

In this project, comparative studies on the content of Glu, Gln, and GABA in the hippocampus were conducted using two chemical models of ASD in rats. *In vivo*, non-invasive MRS and invasive hippocampal microdialysis combined with HPLC analysis of microdialysates were used. In addition, *ex vivo* measurements of amino acid contents in hippocampal homogenates using HPLC or NMR analysis were performed. Some of these changes were gender-dependent. Greater differences in amino acid concentrations as compared to control were observed in males. In addition, more statistically significant differences were observed in both *ex vivo* than in *in vivo* using MRS methods.

Both chemical models of autism used in our studies are based on exposure of rats in their fetal period to teratogenic drugs. The time of forming of the neural tube which is crucial to the development of the Central Nervous System (CNS) appears to be the most sensitive period for such exposure. Rice and Barone (2000) studied early embryos of rats and humans. They concluded that E20–E24 in human embryo corresponds to E9–E11 in rats. In our studies female rats on the 11th day of gestation were administered *per os* one dose of either VPA or THAL, which induced neurodevelopmental changes in their offspring. These non-genetic models have been used in many laboratories to investigate the mechanisms which are potentially involved in development of ASD in humans. Application of these models was helpful in understanding the neurobiology of underlying autistic behavior (Nicolini and Fahnestock, 2018). VPA model of ASD in rats was used for the first time by Rodier et al. (1996). First autism research that studied effects of THAL on rats was performed by a Japanese group in 2002 (Narita et al., 2002). Teitelbaum (2003) studied animals and humans after exposure to THAL during embryo development and confirmed that THAL could be used to model autism for research on primates. The observation that exposure to VPA or THAL during the first trimester of human pregnancy was found to lead to higher incidence of autism in babies (Stromland et al., 1994; Miller et al., 2005; Sadamatsu et al., 2006) was the rationale for the use of animal models of autism using teratogenic drugs. Similar behavioral deficits to those observed in autistic human patients were also observed in rodents prenatally exposed to VPA or THAL (Schneider and Przewlocki, 2005; Schneider et al., 2008)

The weakening of USV emitted by rodent pups separated from their mothers was found by Zippelius and Schleidt (1956) to be the only reliable indicator of pathology similar to autism. The role of the vocalizations in mother-offspring interaction was confirmed multiple times (Wohr and Scattoni, 2013). Kirsten et al. (2012) observed fewer infant USV events at PND 11 in prenatal lipopolysaccharide-administered animal model of autism. In general, unusual or reduced levels of USV were observed in several models of neurodevelopmental disorders (Scattoni et al., 2009), including the mouse model (BTBR T + tf/J mice) of autism (Scattoni et al., 2008). These behavioral deficits and differences are regarded as impairments in social communication (Crawley et al., 2007). We chose this behavioral test, because it can be done as early as PND 9-11. Before USV examination, the animals were tattooed. We realize that our rat model and, hence, our findings have limitations. It relies on not fully adequate animal models with limited validity (Mabunga et al., 2015). Also, we assumed that tattooing has similar impact on control, VPA- and THAL-treated rats. One can imagine that this treatment could cause inflammation, pain and/or discomfort of different degree in analyzed groups. VPA is known to alter the threshold for nociception in rats. It was shown to decrease it in juvenile animals (Dendrinos et al., 2011) and increase it in adult rats (Schneider and Przewlocki, 2005) while, to our best knowledge, it was not studied in pups. Nevertheless, patients with autism reported enhanced sensitivity to thermal pain (Cascio et al., 2008). Moreover, a lifelong method of identification of pups was necessary as non-permanent marking quickly becomes indistinguishable due to pups’ cleaning by the mother and their rapid growth. In the context of pups’ vocalizations, tattooing is the most often used method (Berg et al., 2018). The method was shown to cause little effect in heart rate and blood pressure (Kasanen et al., 2011). Also, at the recording day any wounds caused by ink injection have healed and it seems unlikely that tattooing would leave lasting effects on USV emission 4–5 days later (the day of tattooing and the day of USV recording were separated), i.e., we would not expect any pain-caused effect on vocalization to last that long. Finally, somatosensory cortical coding of pain in rat pups becomes apparent between 2 and 4 weeks of age (Chang et al., 2016). In our investigation, the quantities of USV events were studied using digital sound spectrographic analysis system. In this paper, we present only the quantities of USV events and classified the animals into three subclasses to confirm behavioral differences between control and the autistic animal groups. In the VPA model, more subjects were found to belong to the same subclass as control ones (Figures 4A,B) than in the THAL group. Most of the subjects from the THAL group were located in the first subclass (with fewer number of calls). Subjects in the VPA group were almost equally distributed among all three subclasses. Considering only the results of this behavioral test, we can conclude that greater changes in vocalization occurred in the THAL model than in the VPA model. We tend to attribute these differences to be caused by the effects of different drugs used in modeling of autism, leading to different persistent neurochemical changes in the brains of the test subjects. Notably, opposite effect of VPA and THAL was observed on the level of gene expression in primary neuronal cultures (Zieminska et al., 2016).

The published results concerning Glu/Gln/GABA concentrations in the brain of autistic children, performed with the MRS method differ between individual laboratories (Drenthen et al., 2016; Zheng et al., 2017). Differences in the magnetic field strength of the MRI equipment used in different laboratories for MRS spectroscopy (from 1.5T to 7T) may be the source of the measurement errors causing the differences between the published results (Zheng et al., 2017). In addition, due to the inapplicability of usage of an external reference standard, most of these studies have used the creatine/phosphocreatine (Cr/PCr) as reference signal. However, the assumption that the Cr/PCr ratio in the brain is stable may not be true. It was found that fluctuations of the Cr and PCr concentration in brains of autistic children were higher than in the control ones, contrary to what was previously assumed (Abu Shmais et al., 2012; Ford and Crewther, 2016). In the analysis of MRS data we used database of compounds spectra to determine the presence of tested compounds and calculated their concentrations. This procedure strongly depends on the software and the database. Concentration measurements of compounds with small and overlapped signals always have higher error than measurements obtained from stronger signals. Additionally, the measurement errors are also increased because of the relatively large volume of brain tissue analyzed by MRS containing contaminants like CSF and blood whose amino acid concentrations are different than brain tissue and therefore the obtained results are averages from all these compartments. The results of our current studies using MRS and NMR performed on rat models of autism confirmed higher concentration of Cr in brains of animals in the experimental groups than in the control (data not shown). This lead us to use brain water signal instead of Cr/PCr as reference signal for MRS examinations.

In agreement with the data presented by Page et al. (2006), Joshi et al. (2012) or Hassan et al. (2013) obtained from their studies on humans, we have also detected increased Glu concentration in male rats of both, VPA and THAL groups, however, we have not noticed changes in Gln and GABA levels. Similarly, to our results, no Gln and GABA concentration changes, were reported by Horder et al. (2018) in VPA mouse model. In the brains of autistic children, both the increase (Cochran et al., 2015) and decrease (van Elst et al., 2014) of the Gln level were found. Moreover, most of the reported data (Zheng et al., 2017) indicates decreased level of GABA in humans. In our MRS investigation we observed increased of Glu concentration in female as compared to male control group rats. These results were consistent with the observations of other authors from their studies performed on juvenile rats (Al-Suwailem et al., 2018). Despite the numerous limitations of this method, MRS is still the best and the only method allowing the non-invasive measurement of content of neurotransmitters in the living brain.

Past studies of autism in humans that investigated the glutamatergic dysfunction were mainly focused on evaluation of total glutamate level in brain using MRS (Rojas, 2014). Brain microdialysis method allowed to measure the basal concentrations of glutamate and other neuroactive amino acids in the extracellular space and their fluctuations induced by depolarization. So far microdialysis method have not been used to study autism induced by VPA and THAL. The effectiveness of microdialysis depends on the molecular weight of the test substance as well as on the type of the probe, its length and the flow rate of the dialysis medium (Benveniste, 1989). In studies of the control groups using 2 mm microdialysis probe CMA/11 and medium flow rate of 2 μl/min, Lasley and Gilbert measured basal concentration levels of Glu and GABA to be at 0.48 and 0.02 μM, respectively (Lasley and Gilbert, 2002). Other studies used the same type of the probe but different flow rate (0.6 μl/min) measured basal Glu concentration of 0.99 μM (Schepers et al., 2008) for the control group. In our experiments using the same probe type, we measured median values for basal levels of Glu and GABA in control group to be about 1.5 – 3 times higher than obtained by Lasley and Gilbert (2002). Possibly, slight differences in procedures of HPLC amino acid analyses caused the different results. It is known that due to the high activity of glutamate transporters in the brain, after the KCl depolarization, it is necessary to use inhibitors of glutamate reuptake to observe increase of glutamate concentration in extracellular space (Herrera-Marschitz et al., 1992; Lasley and Gilbert, 2002). In our experiments, we used the microdialysis method without introducing any pharmacological interventions, such as inhibitors of glutamate transporters. We believe that could be the reason that after application of a high concentration of KCl in the microdialysis medium, we did not observe an increase of Glu concentration in the microdialysates (Figure 6). Furthermore, we did not observe gender dependency of concentrations of Glu, Gln or GABA in the dialysates, therefore we presented results without making distinctions for gender (Figure 6). Analysis of samples collected after application of high KCl concentration revealed decreased level of Glu in VPA group. Moreover, we detected lower Gln concentration in both VPA and THAL groups then in the control and also lower within each group as compared to their basal levels. It is a well-known phenomenon that high concentrations of KCl in the microdialysis medium of the rat hippocampus decrease the glutamine level in dialysates by approximately 50% (Millan et al., 1994). It is known that stimulation of rat hippocampus with high potassium added to the microdialysis medium induces a several-fold increase in GABA concentration in the dialysate (Millan et al., 1994). In our experiments, after KCl stimulation, GABA level increased significantly in all tested groups without significant differences between groups. We observed that basal level of Gln in VPA and THAL groups was lower than in the control group (Figure 6). In summary, the changes in Glu/Gln/GABA concentrations in microdialyzates of VPA and THAL groups compared to the control group as detected in this study seem to be consistent with the above described changes in amino acid-mediated neurotransmission in autistic-like animals.

In addition to similar direction of changes in amino acid levels detected by various methods used in this work, significant differences in concentration values were also observed. There are multiple reasons for these differences. In our opinion, great care should be also taken when comparing the results of *in vivo* and *ex vivo* measurements. First of all, for each method different anesthetics were used. Urethane anesthesia, which was employed during microdialysis experiments could potentially interfere with HPLC and NMR data, while volatile anesthetic isoflurane could affect the results of *in vivo* MRS spectroscopy. *Ex vivo* studies demonstrated effects of different concentrations of urethane to inhibit excitatory NMDA and AMPA receptors and potentiates nicotinic, glycine and GABA receptors (Hara and Harris, 2002). However, concentration of 10 mM corresponding to plasma level of urethane in anesthetized animals only modestly affects activities of these channels (Hara and Harris, 2002) and decreases excitability of CA1 neurons by increasing K+ conductance and reducing spontaneous pre-synaptic glutamate release (Tian et al., 2012). Also our control *ex vivo* NMR experiments showed no effect of urethane anesthesia on the Glu, Gln, and GABA content in the rat hippocampus (results not shown). This implies that the urethane mainly affects the activity of ion channels but does not affect the level of neurotransmitters in the brain.

Several studies demonstrated that isoflurane affects the changes in neuroactive amino acid levels in CSF and in plasma similarly as if induced by energy deprivation or posttraumatic brain injury (Berg-Johnsen and Langmoen, 1992; Larsen et al., 1998; Stover et al., 2000, 2004). More recent studies using *in vivo* proton magnetic resonance spectroscopy (1H MRS) showed only minor effects of different doses of isoflurane on cerebral glutamate or glutamate complex (Glx) in mice and dogs (Kulak et al., 2010; Sobbeler et al., 2018). Collectively, this data suggests that different methods of anesthesia do not have a significant effect on measured concentration of Glu, Gln, and GABA levels in rat hippocampus using the *in vivo* MRS method, *ex vivo* HPLC and NMR examinations.

The two methods (*in vivo* and *ex vivo*) used different measurement units; the results of MRS measurements were expressed in mmol/kg wet weight, while data from HPLC and NMR was presented in μmol/mg protein. In addition, different processing and extraction procedures were applied for both *ex vivo* amino acid measurements methods. One of the reasons for the differences in the results between the *ex vivo* measurement methods could be attributed to predicted measurement errors of 10% for NMR and less than 5% for HPLC (Bartolomeo and Maisano, 2006). The size of VOI for the MRS examination was large enough to cover both left and right hippocampus and thus signals from CSF and blood were also measured. Therefore, the measuring error could reach as high as 20–30% (Bhogal et al., 2017). After analysis of the spectra obtained by MRS, NMR and HPLC, we concluded that HPLC and NMR is better than the MRS method for separation of amino acids under-test from other compounds. Both *ex vivo* methods (NMR and HPLC) allowed us to assess total concentration of amino acid in the hippocampus. NMR spectroscopy measured the free compounds in the sample while HPLC detected compound derivatives. Although the same brain homogenate samples were used for both measuring methods, the preparation procedures were different. All of the samples used for the HPLC method were frozen and stored until we performed the measurements, whereas NMR spectroscopy analyses was performed on fresh samples as soon as they were collected. The differences in the sample preparation (fresh vs. frozen and thawed) and extraction for NMR vs. extraction and derivatization of tested amino acids for HPLC could be the cause of some the differences in results obtained by HPLC as compared to NMR method.

It is difficult to assess whether the increase in Glu and GABA content in the rat hippocampus shown in some of our experimental groups using MRS, HPLC or NMR methods (Figures 5, 7, 8) corresponds to strengthening the glutamatergic and GABA-ergic neurotransmission. First of all, the increase in Glu content shown in our studies using MRS, HPLC or NMR applies to the entire hippocampus, without distinguishing between neurons and glial cells, and various intracellular pools. In particular, we do not have data to support the claim that the neurotransmitter pools of Glu and GABA are participating in this increase. Moreover, the results of microdialysis experiments, giving insight into the concentrations of these amino acids in the extracellular space of the hippocampus, were inconclusive. In the VPA and THAL groups, we did not find any significant differences in basal Glu and GABA concentrations in dialysates compared to the control. Moreover, there was no difference between experimental groups in concentration of GABA after KCl stimulation, which does not support the hypothesis of enhancing GABA-ergic neurotransmission. In these studies, we did not use inhibitors of glutamate transporter (see Discussion above). For this reason, it was not possible to observe the increase in the extracellular Glu concentration caused by KCl stimulation, which would reflect the depolarization-dependent release of Glu from the neurotransmitter (vesicular) pool. Instead, we observed a decrease in Glu concentration in the VPA group. Further studies are needed to determine if this effect is due to the increased activity and/or expression of glutamate transporters. It is also not known whether the effect of Glu and GABA level changes contributes to the development of autistic-like behaviors in the studied animals. We observed in PND 30 rats increase of GABA content, if this also happens during the embryonic period then it may lead to stimulation of postsynaptic neurons rather than to their inhibition and consequently to neurodevelopmental disturbances (Owens et al., 1996). These issues are beyond the scope of our study and require further investigations as far as the neurochemical aspects of rat models of autism. Large variability in the results within each of the experimental groups (Figures 6–8) affects the choice of the number of animals for future experiments.

In our study we used two statistical analyses: univariate (ANOVA) and multivariate (OPLS-DA). Both analyses were performed on total concentration values of Glu, Gln, and GABA obtained using each of the measurement methods from both study groups and the control group. Using HPLC method, statistical analysis indicated ten parameters as significantly different, using NMR indicated seven and using MRS only two. Table 1 presents only statistically significant parameters and their direction of changes. The obtained results showed that both univariate and multivariate statistical analyses generated the same final results. The advantage of using OPLS-DA is that the analysis can be carried out on all data parameters and all groups simultaneously (in our case on three experimental groups). OPLS-DA analysis of Glu, Gln, and GABA concentrations allowed to build models, to perform model validation and to identify the putative biomarkers. OPLS-DA method is less time consuming as compared to univariate methods in particular for large number of parameters.

**Table 1:** Direction of total concentrations changes of Glu, Gln, and GABA and direction changes of USV behavioral test results as compared to control in rat models of autism (VPA or THAL treatment), taking into the account the gender of animals (F, females; M, males; F + M, whole group).

	USV	MRS	NMR	HPLC
VPA – M		Glu ↑	Gln ↑ GABA ↑	Glu ↑ Gln ↑ GABA ↑
THAL – M	↓	Glu ↑	Gln ↑	Glu ↑ Gln ↑ GABA ↑
VPA – F			Gln ↓	
THAL – F	↓			
VPA – M + F	↓		GABA ↑	Glu ↑ GABA ↑
THAL – M + F	↓		Glu ↑ Gln ↑	Glu ↑ GABA ↑


## Conclusion

Both animal models of autism used in this project have demonstrated their applicability in the studies on disturbances of neuroactive amino acid homeostasis in the rat brain. We observed sex dependency of analyzed parameters in both experimental groups and in the control group as well. We observed statistically significant differences between autistic-like animals and control ones for more parameters in male then in female rats. Extensive *ex vivo* analysis using HPLC and NMR revealed more statistically significant differences in more parameters than the *in vivo* approach using MRS. For all methods used, the values of measured changes were in the same direction. OPLS-DA analysis confirmed that both animal models of autism tested here are capable of differentiating diseased animals from control ones and can be used to trace biochemical changes in the brain. We also observed higher homogeneity of each study group data in THAL vs. control model than in VPA vs. control model.

## Author Contributions

EZ: originated the research concept and contributed to the design of the study, prepared animal models for experiments, prepared samples for NMR measurements, contributed to interpreting the results and preparation of the figures, and participated in the draft of the manuscript. BT: contributed to the design of the study, measured and analyzed NMR spectra, performed the statistical analysis of all experimental data, contributed to interpreting the results and preparation of the figures, and participated in the draft of the manuscript. DD: performed microdialysis of rat hippocampus and collected samples for HPLC analysis. WH: contributed to the design of the study, performed HPLC analysis, and participated in the draft of the manuscript. RP and RF: contributed to the design of the study, performed behavioral studies, and participated in the draft of the manuscript. JO: contributed to the design of the study, performed and analyzed MRS spectra, and participated in the draft of the manuscript. MG: measured and analyzed NMR spectra. JL: contributed to the design of the study, interpreted the results of the experiments, and participated in the draft of the manuscript. All authors revised and approved the final version of the manuscript.

## Conflict of Interest Statement

The authors declare that the research was conducted in the absence of any commercial or financial relationships that could be construed as a potential conflict of interest.
